# ﻿*Yushaniadezhui* (Poaceae, Bambusoideae), a new bamboo species from Yunnan, China

**DOI:** 10.3897/phytokeys.246.130177

**Published:** 2024-08-16

**Authors:** Yu-Xiao Zhang, Chao Zhang, Ru-Li Zhang, Hu-Gang Zhao

**Affiliations:** 1 Yunnan Academy of Biodiversity, Southwest Forestry University, Kunming, Yunnan 650224, China Southwest Forestry University Kunming China; 2 College of Landscape Architecture and Horticulture Sciences, Southwest Forestry University, Kunming, Yunnan 650224, China Southwest Forestry University Kunming China

**Keywords:** Limestone montane area, long-necked rhizome, new taxon, temperate woody bamboos

## Abstract

A new bamboo species, *Yushaniadezhui*, from Kunming, Yunnan, China is described and illustrated in this paper. The new species used to be misidentified as *Y.polytricha*. Based on careful comparison of morphological features and molecular phylogeny evidence, we confirmed its identity as a new member of the genus *Yushania*. *Yushaniadezhui* resembles *Y.maculata*, *Y.polytricha* and *Y.weixiensis* in several aspects, such as culm height and branch complement structure. However, the glabrous culm leaf sheaths and internodes, the absence of auricles and oral setae on most foliage leaves, except the one-year-old foliage leaves, the pubescence on the adaxial surface of the one-year-old foliage leaves and its limestone habitat preference can readily distinguish this new species from its related taxa. Moreover, we emphasise that individuals from various populations and molecular markers with different inheritance patterns for phylogeny reconstruction should be included in new species discovery, especially in plant groups with complex evolutionary histories.

## ﻿Introduction

*Yushania* P.C. Keng is a genus diversified within the Hengduan Mountains region and followed by subsequent spreading outside this region (Ye et al. 2019). It belongs to the subtribe Thamnocalaminae of the tribe Arundinarieae (Poaceae, Bambusoideae) (Zhang et al. 2020) and consists of more than 90 species (Vorontsova et al. 2017; Shi et al. 2022). Taxa of *Yushania* are usually distributed in mountainous areas above an elevation of 1000 m and most of them are narrowly endemic to a certain region (Li et al. 2006). Due to the special habitat and distribution pattern, new species of *Yushania* are continuously being described recently from different mountains, such as *Y.tongpeii* D.Z. Li, Y.X. Zhang & E.D. Liu (Zhang et al. 2019), *Y.longshanensis* D.Z. Li & X.Y. Ye (Ye et al. 2021b), *Y.stoloniforma* D.Z. Li & X.Y. Ye (Ye et al. 2021b), *Y.doupengshanensis* Y.Y. Zhang et N.H. Xia (Zhang and Xia 2021), *Y.tongziensis* N.H. Xia, Y.Y. Zhang & G. Xie (Zhang et al. 2023).

There are two *Yushania* species recorded in Kunming, Yunnan, China, i.e. *Y.maculata* T.P. Yi and *Y.polytricha* T.P. Yi (Sun et al. 2003). The type specimen of *Y.polytricha* was collected at the Qiongzhu Temple in the west of Kunming (Yi 1986). During field investigations in recent years, we found a species of *Yushania* occurring in western and northern Kunming, sometimes sympatric with *Y.maculata* and *Y.polytricha*. This species has been misidentified as *Y.polytricha* for many years in floristic treatments and community ecology papers. The short and thin culms of this species resemble *Y.polytricha*, but the branches, auricles of culm leaves and foliage leaves and hairs on the culms, culm leaves and foliage leaves differ significantly from those of *Y.polytricha*. In order to clarify the identity of this species, we performed several field surveys at different times and localities during 2023. We finally confirm that it is new to science based on morphological and molecular evidence and describe it in this paper.

## ﻿Materials and methods

### ﻿Field investigation, morphological feature observation and comparison

Several field investigations were carried out during late June to late August 2023 in Kunming, including Changchong Hill and Xiaoshao Village of Ciba Town, Haikou Forest Farm of West Hill District and Xianfeng of Xundian County. Specimens were collected in the field and deposited at the Herbarium of the Kunming Institute of Botany, Chinese Academy of Sciences. Young and healthy foliage leaves were sampled and dried in silica gel for the molecular experiment. Morphological features of the new species were observed and recorded in the field and the lab. Comparison of morphological characteristics amongst sympatric or morphologically similar taxa in *Yushania* (i.e. *Y.maculata*, *Y.polytricha*, *Y.weixiensis* T.P. Yi) was performed, based on field observation, specimens and literature (e.g. Li et al. (2006)).

### ﻿DNA extraction, sequencing and assembly

Total genomic DNA was extracted from the silica gel-dried leaves using TIANGEN Magnetic Plant Genomic DNA Kit (TIANGEN, Beijing, China). All procedures were performed according to the manufacture’s instruction. DNA concentration, integrity and purity were examined using the Agilent 5400 system (Agilent, USA). Subsequently, the DNA samples were fragmented by sonication to a size of 350 bp. Then DNA fragments were end-polished, A-tailed and ligated with the full-length adapter for Illumina sequencing, followed by further PCR amplification. PCR products were purified by AMPure XP system (Beverly, USA). Library quality was assessed on the Agilent 5400 system (Agilent, USA)and quantified by QPCR (1.5 nM). The qualified libraries were pooled and sequenced on Illumina NovaSeq 6000 platform (Illumina, San Diego, CA, USA) with PE150 strategy. Finally, a total of 2 GB data per sample was generated. All those experiments were carried out at Novogene Bioinformatics Technology Co., Ltd (Beijing, China).

After quality control of the raw data by Fastp 0.23.2 (Chen et al. 2018) with default parameters, all paired reads were extracted for plastid assembly using GetOrganelle 1.7.2 (Jin et al. 2020) and the plastome sequence of *Y.niitakayamensis* (Hayata) P.C. Keng (MN310560.1) was used as a reference. Subsequently, the de novo assembled maps were conducted using Bandage 0.9.0 (Wick et al. 2015) to visualise the complete plastomes. The assembled plastome sequences were aligned and checked collinearly by Mauve 2.4.0 (Darling et al. 2004) with default settings in order to make sure the structure and direction were consistent with the reference plastome. The plastid genes were annotated using CPGAVAS2 online (Shi et al. 2019) based on the annotation of *Y.niitakayamensis* with manual adjustment in Geneious Prime 2022.0.1 (Kearse et al. 2012). In addition, the ribosomal DNA sequence of *Oryzasativa* cultivar TN1 (KM036285.1) (Guo et al. 2021) was used as a reference to assemble and annotate the entire nrDNA sequences in Geneious Prime 2022.0.1.

### ﻿Phylogenetic analysis

To confirm the phylogenetic affinity of the new species, plastome and nrDNA sequences of representatives of the tribe Arundinarieae were used to reconstruct the phylogenetic trees, based on the results of Guo et al. (2021). A total of 30 sequences from 26 species were selected, including eight newly-sequenced plastome and nrDNA sequences, respectively (Table 1). The plastome and nrDNA sequences were aligned using MAFFT 7.520 (Katoh and Standley 2013). The TPM3+F+I+G4 model for plastomes and the TPM3+F+I+G4 model for nrDNA were selected using ModelFinder 2.2.5 (Kalyaanamoorthy et al. 2017), based on the Bayesian Information Criterion (BIC). Subsequently, Maximum Likelihood (ML) analyses were performed using IQ-TREE 2.2.5 (Nguyen et al. 2015) with 1000 ultrafast bootstrap replicates and SH-aLRT test (Hong et al. 2022). Bayesian Inference (BI) was conducted in MrBayes 3.2.7a (Ronquist et al. 2012) and the GTR+I+G model was selected by jModelTest 2.1.7 (Darriba et al. 2012) using BIC. Markov Chain Monte Carlo (MCMC) simulations were run for 1,000,000 generations, with a sampling every 1000 generations. The initial 25% of generations were discarded as burn-in. A 50% majority-rule consensus tree was constructed when the average standard deviation of split frequencies was below 0.01.

**Table 1.** Voucher information and GenBank accession numbers for plant materials used in this study.

Taxon	Voucher information	GenBank accession No. / source	
		plastome	nrDNA
**Ingroup**			
*Ampelocalamusactinotrichus* (Merrill & Chun) S.L. Chen, T.H. Wen & G.Y. Sheng	ZXZ151102 (KUN)	Guo et al. (2021)	Guo et al. (2021)
*Chimonocalamuscibarius* T.P. Yi & J.Y. Shi	YD04 (KUN)	Guo et al. (2021)	Guo et al. (2021)
*Chimonocalamusfimbriatus* Hsueh & T.P. Yi	GC141-3 (KUN)	Guo et al. (2021)	Guo et al. (2021)
*Fargesiaacuticontracta* T.P. Yi	YXY266-1 (KUN)	Guo et al. (2021)	Guo et al. (2021)
*Fargesiafrigidis* T.P. Yi	ZXZ11023 (KUN)	Guo et al. (2021)	Guo et al. (2021)
*Fargesiamelanostachys* (Handel-Mazzetti) T.P. Yi	YXY145-3 (KUN)	Guo et al. (2021)	Guo et al. (2021)
*Fargesianivalis* T.P. Yi & J.Y. Shi	YXY125-2 (KUN)	Guo et al. (2021)	Guo et al. (2021)
*Gaoligongshaniamegalothyrsa* (Handel-Mazzetti) D.Z. Li, Hsueh& N.H. Xia	GC120-5 (KUN)	Guo et al. (2021)	Guo et al. (2021)
*Hsuehochloacalcarean* (C. D. Chu & C. S. Chao) D.Z. Li & Y.X. Zhang	GC82 (KUN)	Guo et al. (2021)	Guo et al. (2021)
*Indocalamushirtivaginatus* H.R. Zhao & Y.L. Yang	GC94-6 (KUN)	Guo et al. (2021)	Guo et al. (2021)
*Indocalamuslatifolius* (Keng) McClure	GC58-2 (KUN)	Guo et al. (2021)	Guo et al. (2021)
*Indocalamustessellatus* (Munro) P.C. Keng	GC88-8 (KUN)	Guo et al. (2021)	Guo et al. (2021)
*Indosasacrassiflora* McClure	GY15039-B (KUN)	Guo et al. (2021)	Guo et al. (2021)
*Phyllostachysincarnata* T.H. Wen	ZLN-2011035 (KUN)	Guo et al. (2021)	Guo et al. (2021)
*Pleioblastusamarus* (Keng) P.C. Keng	G14151-B (KUN)	Guo et al. (2021)	Guo et al. (2021)
*Pleioblastusfortune* (Van Houtte) Nakai	GC33-2 (KUN)	Guo et al. (2021)	Guo et al. (2021)
*Pseudosasaguanxianensis* T.P. Yi	GC62-3 (KUN)	Guo et al. (2021)	Guo et al. (2021)
*Shibataeakumasaca* (Zollinger ex Steudel) Makino ex Nakai	GC31-3 (KUN)	Guo et al. (2021)	Guo et al. (2021)
*Yushaniabrevipaniculata* (Handel-Mazzetti) T.P. Yi	YXY043 (KUN)	Guo et al. (2021)	Guo et al. (2021)
*Yushaniadezhui* Y.X.Zhang &R.L.Zhang	CCS03 (KUN)	OR750780	OR760053
*Yushaniadezhui* Y.X.Zhang &R.L.Zhang	DSTQ02 (KUN)	OR750779	OR760052
*Yushaniadezhui* Y.X.Zhang &R.L.Zhang	XS01 (KUN)	OR750778	OR760051
*Yushaniadezhui* Y.X.Zhang &R.L.Zhang	ZSC03 (KUN)	OR750777	OR760050
*Yushanialongiuscula*T.P. Yi	YXY154-1 (KUN)	Guo et al. (2021)	Guo et al. (2021)
*Yushaniamaculata*T.P. Yi	DSTQ01 (KUN)	OR750784	OR760057
*Yushanianiitakayamensis* (Hayata) P.C. Keng	12321 (KUN)	Guo et al. (2021)	Guo et al. (2021)
*Yushaniapolytricha*T.P. Yi	QZS001 (KUN)	OR750783	OR760056
*Yushaniashuichengensis* T.P. Yi & L. Yang	LPS15 (KUN)	OR750782	OR760055
*Yushaniashuichengensis* T.P. Yi & L. Yang	LPS22 (KUN)	OR750781	OR760054
**Outgroup**			
*Chusqueaculeou* E. Desvaux	GZH-089 (KUN)	Guo et al. (2021)	Guo et al. (2021)

## ﻿Results

The new species (*Yushaniadezhui*), *Y.maculata* and *Y.polytricha* all have solid rhizome necks, while *Y.weixiensis* possesses hollow ones. The culm height of *Y.dezhui*, *Y.polytricha* and *Y.weixiensis* is usually less than 2 m, whereas *Y.maculata* is more than 2 m tall. The internode and sheath scar of *Y.dezhui* are glabrous, which is different from the other three related species, which have at least some hairs on the internode and sheath scar. Dark purple-brown spotted culm leaf sheaths and purple oral setae differentiate *Y.maculata* from *Y.dezhui*. Auricles and oral setae of culm leaves and foliage leaves of *Y.dezhui* are usually absent, while *Y.polytricha* has conspicuous auricles and oral setae. Moreover, a different branch number per node and setae on culm leaves and foliage leaves can also distinguish *Y.dezhui* from *Y.polytricha* (see Table 2 for details). Although *Y.dezhui* and *Y.weixiensis* both have relatively short culms, usually glabrous culm leaves without auricles and oral setae and similar branch numbers, characters of rhizome neck, internode and foliage leaf can differentiate these two species (see Table 2 for details).

**Table 2.** Morphological comparison of *Yushaniadezhui* and related species.

	*Y.dezhui*	*Y.maculata*	*Y.polytricha*	*Y.weixiensis*
Rhizome neck	5–12 mm in diameter, solid	5–10 mm in diameter, solid	3–8 mm in diameter, solid	7–10 mm in diameter, hollow
Culm	0.4–1.8 m tall, 1–5 mm in diameter	2–3.5m tall, 0.8–1.5 cm in diameter	1–2 m tall, 3–8 mm in diameter	1–2 m tall, 3–10 mm in diameter
Internode	Subsolid, purple-spotted initially, slightly white powdery below nodes	Hollow, initially densely white powdery, with grey or light yellow setae	Solid, initially densely purple spotted, thinly white powdery and yellow-brown setulose below nodes	Hollow, initially white powdery, white-grey setulose
Sheath scar	Corky, glabrous	Prominent, initially densely brown setose	Prominent, initially densely yellow-brown retrorse-setose	Prominent, initially yellow-brown setose
Branch complement	3–12	7–12	1–5	3–7
Culm leaf	Tardily deciduous, ca. 1/2 as long as the internode; sheaths glabrous abaxially, occasionally white pubescent at the base	Persistent, dark purple-brown spotted, ca. 1/3 as long as internodes, mainly glabrous, but sparsely brown setose at the base	Persistent, ca. 2/3 as long as internodes, yellow-brown setose	Persistent, glabrous or sparsely yellow-brown setose
Culm leaf auricle & oral setae	Auricles linear or absent, usually present on upper culm leaves; oral setae several when auricles present	Auricles absent; oral setae 3–5, erect, purple	Auricles purple, falcate; oral setae many, radiating, yellow-brown	Auricles and oral setae absent
Foliage leaf sheath	Glabrous, green, purple or purple-green, 2.5–5.2 cm long, margins glabrous	Glabrous, 4.5–6 cm long, margins glabrous	Densely brown setose, margins densely yellow ciliate	Glabrous, 1.2–2.7 cm long, margins glabrous
Foliage leaf auricle & oral setae	Auricles usually present on the one-year-old foliage leaves, linear, green or purple; oral setae several when auricles present	Auricles absent; oral setae 3–5, Erect, purple	Auricles purple, falcate or elliptic; oral setae several, erect or radiating, yellow-brown	Auricles absent; oral setae 3–5, yellow
Foliage leaf blade	2.5–12.5 × 0.5–1.3 cm, pubescent adaxially for the one-year-old blades, glabrous abaxially, secondary veins 2–4 paired	9–15 × 0.9–1.1 cm, glabrous, secondary veins 4-paired	9–21 × 1.2–2.5 cm, abaxially grey pubescent, secondary veins 4–6-paired	3.4–7 × 0.3–0.6 cm, glabrous, secondary veins 2- or 3-paired

The length of the eight newly-sequenced plastomes ranged from 139599 bp (*Y.shuichengensis* T.P. Yi & L. Yang LPS15) to 139653 bp (the new species *Y.dezhui* XS01), with a consistent GC content of 38.90%. They showed a typical quadripartite circular structure, which consisted of a pair of inverted repeat regions (IR), one large single copy region (LSC) and one small single copy region (SSC). The plastome length of the new species varied from 139616 bp (ZSC03) to 139653 bp (XS01). The newly-assembled eight nrDNA sequences had a length from 8337 bp in *Y.shuichengensis* (LPS22) to 9010 bp in *Y.polytricha* (QZS001) and comprised of 18S (1811 bp), ITS1 (215–216 bp), 5.8S (165 bp), ITS2 (215–217bp), 26S (3392 bp) and an intergenic spacer (IGS, 2539–3212 bp). For the new species, individuals ZSC03 and CCS03 had the same length (8695 bp) of nrDNA sequences, while the length of the individual XS01 was four bp longer than the individual DSTQ02 (216 bp vs. 217 bp in the ITS2 region and 2898 bp vs. 2893 bp in the IGS region).

**Figure 1.**
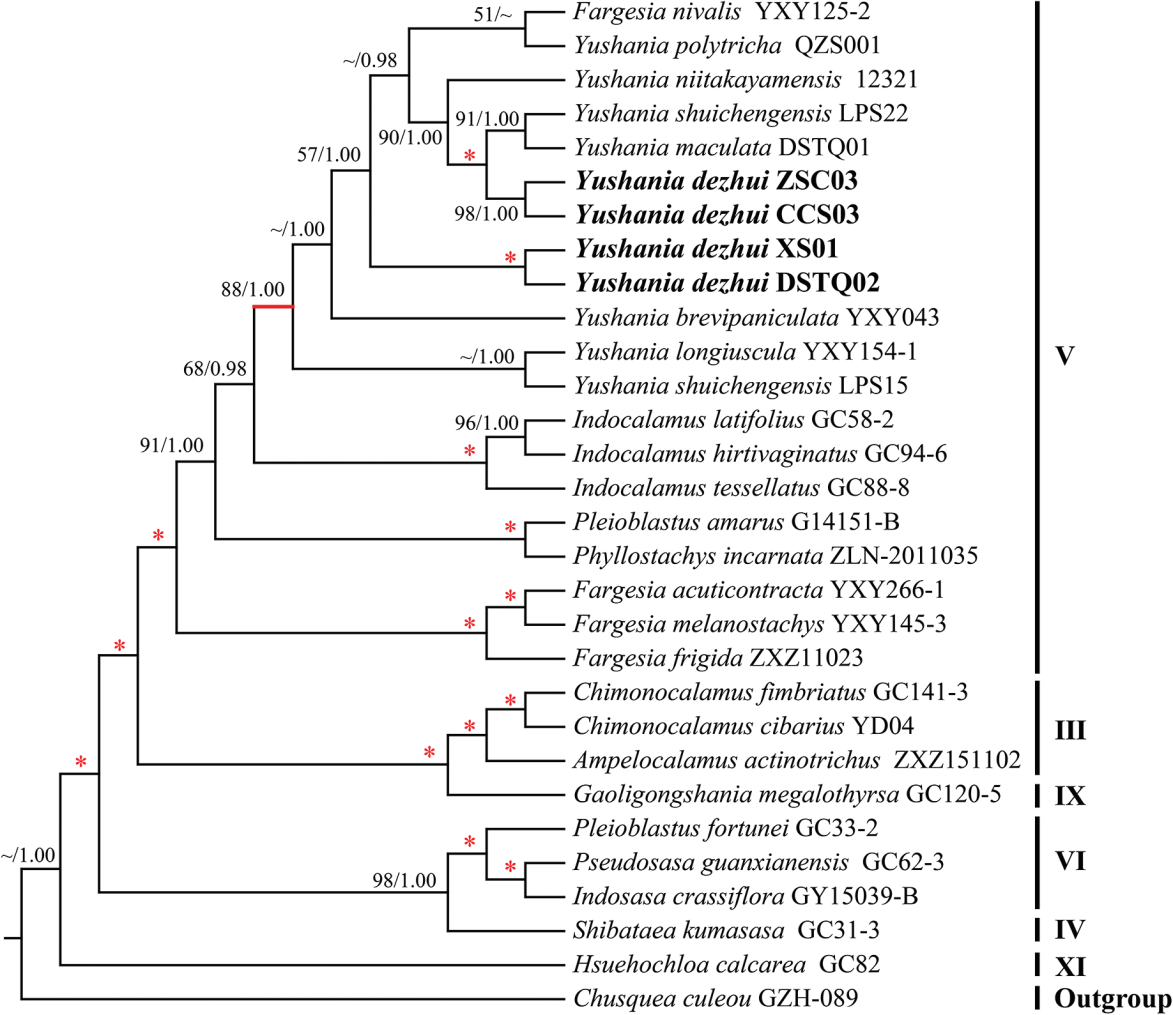
Maximum Likelihood phylogenetic tree reconstructed from plastome sequences of the tribe Arundinarieae. Numbers along branches indicate the Maximum Likelihood bootstrap values (MLBP) and Bayesian posterior probabilities (BI). * means MLBP/BI=100/1.00. The Roman numbers on the right of this tree correspond to those lineages recovered in previous studies (Yang et al. 2013). The branch in red (MLBP/BI = 88/1.00) denotes the clade containing *Yushaniadezhui* and its relatives.

After alignment, the plastome sequences had a total length of 143257 bp, including 3994 variable sites and 783 parsimony informative sites. In the plastome tree, six lineages were recovered with high support, i.e. clades III-VI, clades IX and XI (Fig. 1). The four samples of the new species were nested within the clade V and formed two subclades. One subclade consisted of individuals ZSC03 and CCS03 (MLBP/BI = 98/1.00) and the other clade included individuals XS01 and DSTQ02 (MLBP/BI = 100/1.00). The new species had close relationships with *Fargesianivalis* T.P. Yi & J.Y. Shi, *Yushaniamaculata*, *Y.niitakayamensis* (Hayata) P.C. Keng, *Y.polytricha* and *Y.shuichengensis* (LPS22).

**Figure 2.**
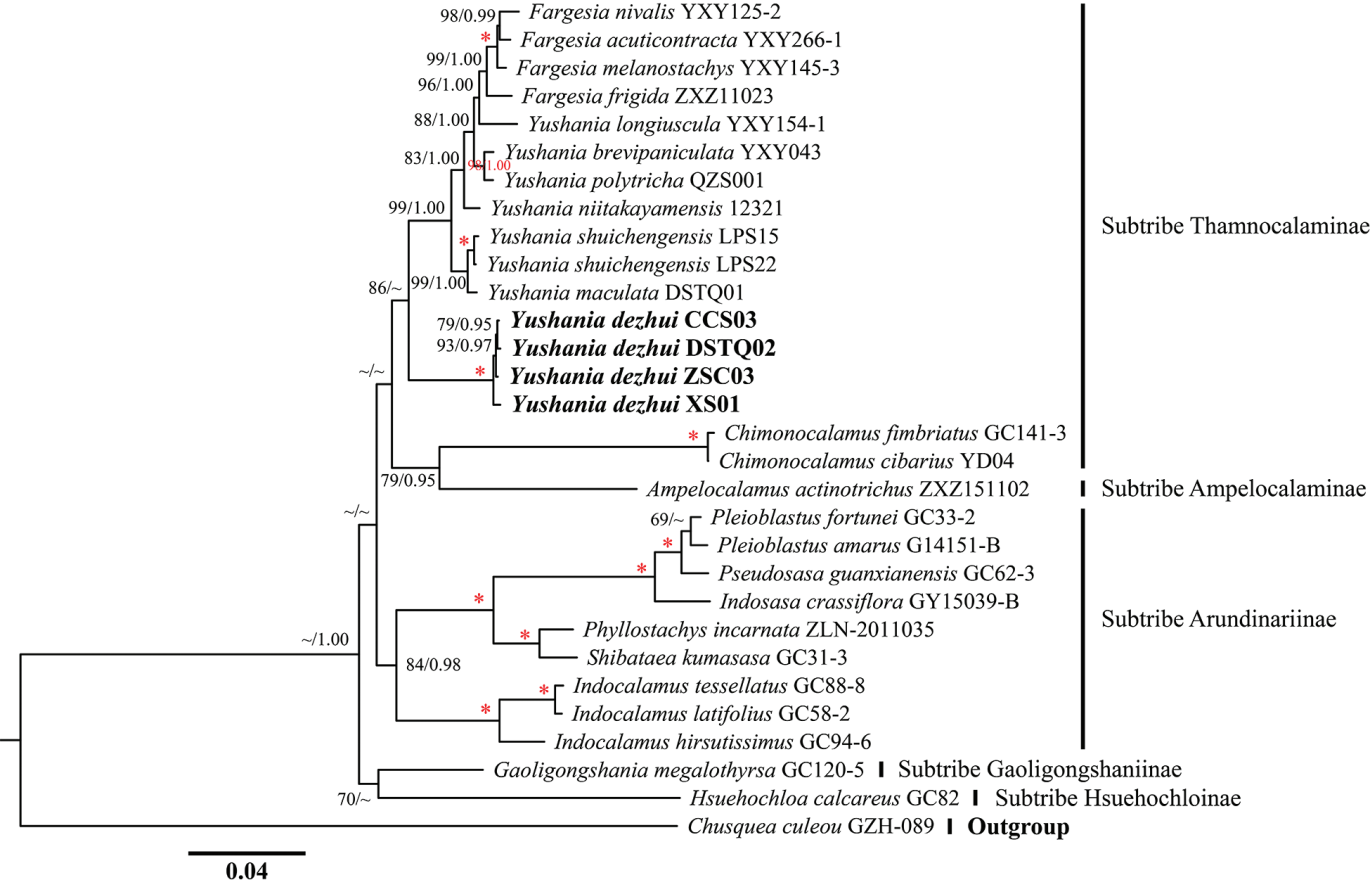
Phylogenetic tree reconstructed from nrDNA sequences of the tribe Arundinarieae by using the Maximum Likelihood method. Numbers along branches indicate the Maximum Likelihood bootstrap values (MLBP) and Bayesian posterior probabilities (BI). * means MLBP/BI = 100/1.00.

The aligned length for nrDNA sequences was 13004 bp with 2571 variable sites and 1502 parsimony informative sites. In the nrDNA topologies, five subtribes were revealed, including subtribes Ampelocalaminae, Arundinariinae, Gaoligongshaniinae, Hsuehochloinae and Thamnocalaminae (Fig. 2). All the four individuals of the new species were grouped into a clade (MLBP/BI = 100/1.00) and nested in the subtribe Thamnocalaminae.

### ﻿Taxonomic treatment

#### ﻿ Yushania dezhui

Taxon classificationPlantaePoalesPoaceae

Y.X.Zhang & R.L.Zhang sp. nov.

794C4E60-7A5B-5D35-80A5-4F059DAE22BB

urn:lsid:ipni.org:names:77346804-1

Figs 3 , 4 , 5

##### Diagnosis.

*Yushaniadezhui* resembles *Y.maculata* T.P. Yi, *Y.polytricha* Hsueh & T.P. Yi and *Y.weixiensis* T.P. Yi, but differs in having rhizome necks much thicker than culms, glabrous culm leaf sheaths, internodes and sheath scars, auricles and oral setae of most foliage leaves usually absent and the one-year-old foliage leaves with pubescence adaxially.

**Figure 3.**
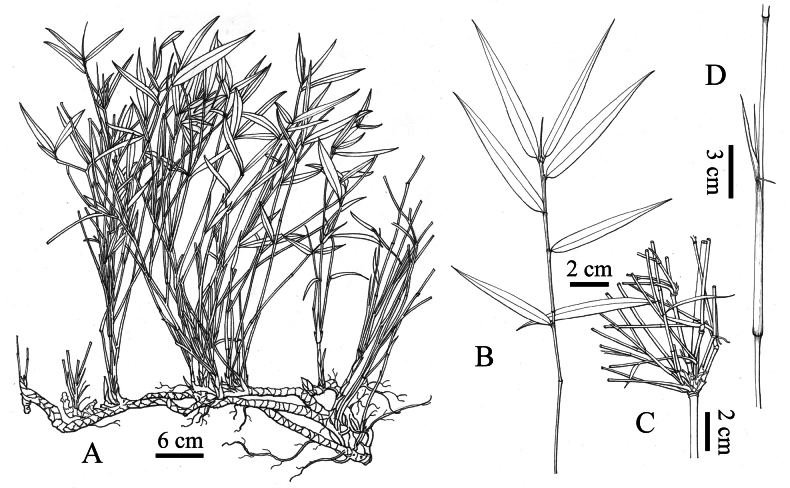
*Yushaniadezhui* Y.X.Zhang & R.L.Zhang **A** clump **B** foliage leafy branch **C** branches **D** internode and culm leaf.

##### Type.

China • Yunnan: Kunming City, Panlong District, Ciba Town, Changchong Hill; 25°07'28.87"N, 102°42'17.41"E; 2226 m a.s.l.; 22 August 2023; *Y.X. Zhang et al. CCS03* (holotype, KUN!; isotype, IBSC!).

**Figure 4.**
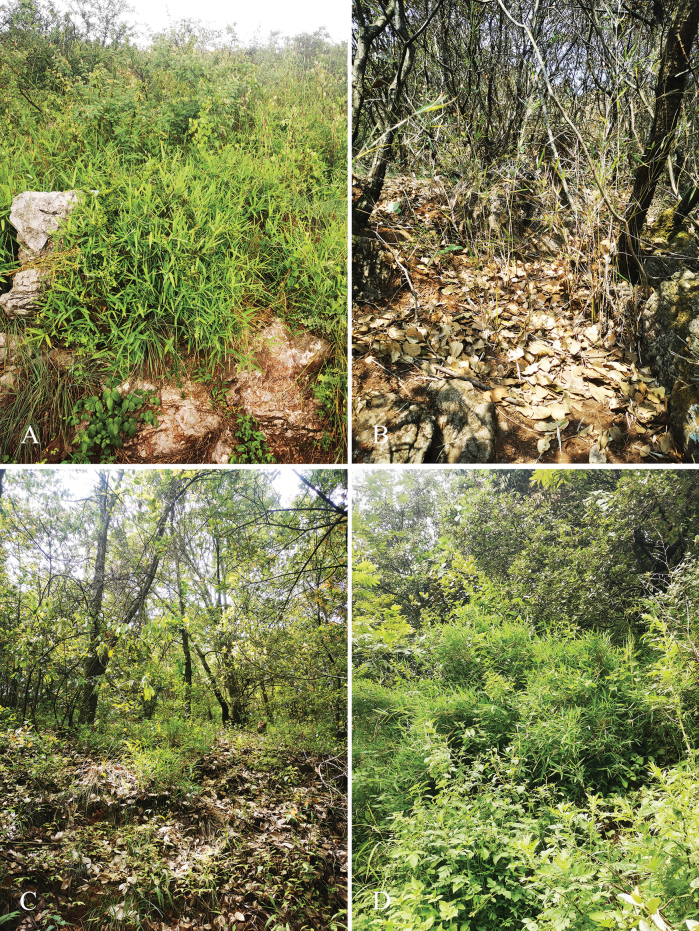
Habitats of *Yushaniadezhui* Y.X.Zhang & R.L.Zhang **A** Changchong Hill, voucher No. CCS03 **B** Xiaoshao Village, voucher No. XS02 **C** Haikou, voucher No. ZSC03 **D** Xundian, voucher No. DSTQ02. Photos by Yu-Xiao Zhang.

##### Description.

Rhizomes pachymorph, necks 2–30 cm long, 0.5–1.2 cm in diameter, internodes 0.2–1 cm long, solid. Culms diffuse, 0.4–1.8 m tall, 1–5 mm in diameter, subsolid; internodes 2.5–21.5 cm long, terete, purple-spotted initially, slightly white powdery below the nodes; nodes slightly prominent; sheath scars corky. Branches 3–12, slender and equal. Culm leaves tardily deciduous, ca. 1/2 as long as the internode; sheaths glabrous abaxially, green or purple apically, margins ciliate, occasionally white pubescent at the base; auricles linear or absent, usually present on upper culm leaves; oral setae several when auricles present; ligules truncate, 1–2 mm tall, green or purple, margins ciliate or not; blades linear, recurved, glabrous, 0.3–2.3 cm long. Foliage leaves 3–5 per ultimate branch; sheaths glabrous, green, purple or purple-green, 2.5–5.2 cm long, margins glabrous; auricles and oral setae usually absent, except present on the one-year-old foliage leaves, auricles linear, green or purple; ligules truncate, 1–2 mm tall, green or purple; blades 2.5–12.5 × 0.5–1.3 cm, pubescent adaxially for the one-year-old blades, glabrescent later, glabrous abaxially, secondary veins 2–4-paired, transverse veins conspicuous, margins serrate on both sides. Inflorescence unknown.

**Figure 5.**
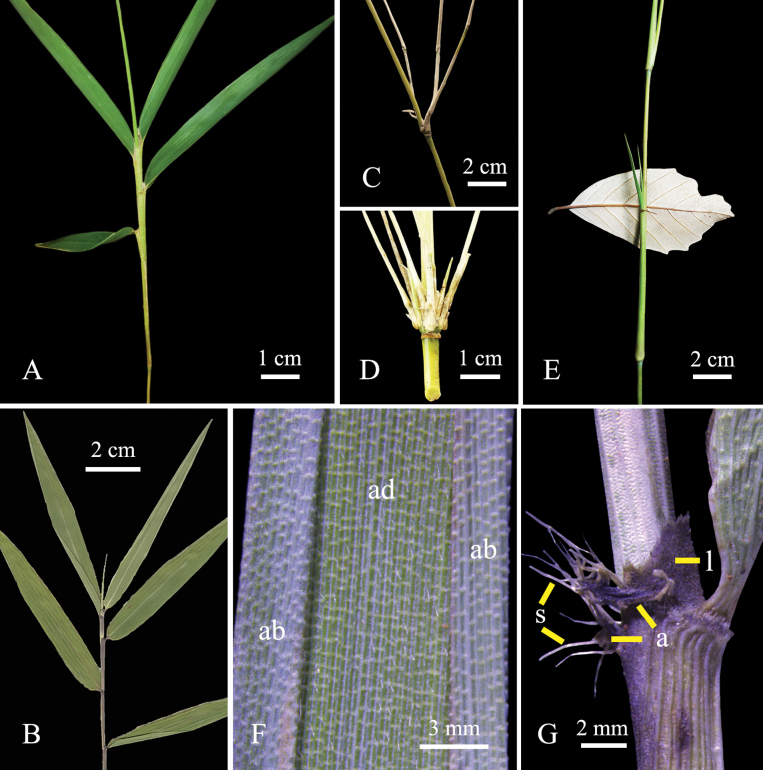
*Yushaniadezhui* Y.X.Zhang & R.L.Zhang **A** foliage leaves with green sheath **B** foliage leaves with purple sheath **C, D** branches **E** internode with culm leaf **F** the one-year-old foliage leaf blade, showing the pubescent adaxial epidermis, ab = abaxial epidermis, ad = adaxial epidermis **G** auricles, oral setae and ligule of the one-year-old foliage leaf, a = auricles, l = ligule, s = oral setae. Photos **A–E** by Yu-Xiao Zhang **F**, **G** by Shun-Shun He.

##### Phenology.

New shoots June to August.

##### Distribution and habitat.

This new species is found in Kunming, Yunnan, China. It usually occurs in limestone montane areas at elevations of 2100–2400 m in the understorey of forests of *Quercuslongispica* (Handel-Mazzetti) A. Camus, *Castanopsisdelavayi* Franchet or *Cupressus* sp.

##### Etymology.

The epithet honours Professor De-Zhu Li of Kunming Institute of Botany, Chinese Academy of Sciences, who has made great contributions to the taxonomy, molecular phylogeny, evolution and biogeography of bamboos and promoted the collaboration of bamboo research around the world.

##### Chinese name.

Dé Zhū Yù Shān Zhú (Chinese pronunciation), 德铢玉山竹 (Chinese characters).

##### Additional specimens examined (paratypes).

China – Yunnan Province, Kunming City • Panlong District, Ciba Town, Changchong Hill; 25°7'31.85"N, 102°42'25.13"E; 2211 m a.s.l.; 26 June 2023; *Y.X. Zhang & C. ZhangCCS01* (KUN); • same collection data as for preceding; 25°6'55.28"N, 102°42'5.67"E; 2314 m a.s.l.; 27 August 2023; *R.L. Zhang et al.CCS04* (KUN); • same collection data as for preceding; 25°7'6.86"N, 102°42'9.98"E; 2310 m a.s.l.; 27 August 2023; *R.L. Zhang et al*. *CCS05* (KUN); • Panlong District, Ciba Town, Xiaoshao Village; 25°11'12.22"N, 102°44'41.14"E; 2243 m a.s.l.; 27 June 2023; *Y.X. Zhang & C. Zhang XS01* (KUN); • same collection data as for preceding; 25°11'10.32"N, 102°44'43.1"E; 2266 m a.s.l.; 27 June 2023; *Y.X. Zhang & C. Zhang* XS02 (KUN); • Xishan District, Haikou Forest Farm; 24°50'50.09"N, 102°36'19.75"E; 2147 m a.s.l.; 18 August 2023; *Y.X. Zhang & R.L. Zhang ZSC02*, *ZSC03* (KUN); • same collection data as for preceding; 2106 m a.s.l.; 25 July 2020; *H. Peng PHHK112* (KUN); • Xundian County, Xianfeng Town; 25°31'12.79"N, 103°04'28.80"E; 2429 m a.s.l.; 21 August 2023; *Y.X. Zhang et al. DSTQ02* (KUN).

## ﻿Discussion

The branch number per node of the new species *Yushaniadezhui* is 3–12, which is similar to *Y.maculata* and *Y.weixiensis*. In *Flora Reipublicae Popularis Sinicae* (Yi 1996), *Yushania* was divided into two sections, i.e. section Brevipaniculatae T. P. Yi and section Yushania. Taxa of the former section usually possess many branches per node, whereas species of the latter one have solitary branch or one branch on lower nodes and 3–5(-8) on middle and upper nodes. Therefore, we place *Y.dezhui* into section Brevipaniculatae. The most unique morphological character of *Y.dezhui* is the adaxial epidermis with pubescence for the one-year-old foliage blades. Up to now, there are no more than 20 bamboo species with hairy adaxial epidermis of foliage blades recorded in China, such as *Bambusapolymorpha* Munro, *Yushaniaqiaojiaensis* Hsueh & T. P. Yi (Li et al. 2006; Shi et al. 2022). Besides those morphological features (Table 2) that can differentiate *Y.dezhui* from sympatric species *Y.maculata* and *Y.polytricha* and morphologically similar species *Y.weixiensis*, the habitat preference is another diagnostic character. The new species *Y.dezhui* usually occurs in limestone montane areas from 2100 m to 2400 m a.s.l., while *Y.maculata* and *Y.weixiensis* are usually distributed on non-limestone shady slopes and at a slightly higher elevation than the new species (2200–3500 m) and *Y.polytricha* occurs at elevations from 1900 m to 1950 m (2000 m) (Li et al. 2006; Shi et al. 2022).

In the plastome phylogenetic analyses, the recovered six main lineages, i.e. clades III–VI, clades IX and XI, were consistent with previous studies (Guo et al. 2021). The relationships amongst the five subtribes in the nrDNA tree were not well resolved, which was also consistent with Guo et al. (2021). The four individuals of *Yushaniadezhui* were grouped into two subclades in the plastome tree (Fig. 1), whereas they formed a clade with high support (100%) in the nrDNA tree (Fig. 2). A similar situation applied to *Y.shuichengensis*. Discordance between plastome and nuclear gene trees has been recovered in the genus *Yushania* and other genera, even in the tribe Arundinarieae (e.g. Zhang et al. (2012); Yang et al. (2013); Guo et al. (2019); Guo et al. (2021); Ye et al. (2021a)). The cytonuclear incongruence was mostly caused by the complex evolutionary history of those taxa, including hybridisation, introgression, incomplete lineage sorting and so on. Ye et al. (2021a) revealed the reticulate evolutionary history of the genera *Fargesia* Franchet and *Yushania*, based on genome skimming and double digest restriction-site-associated DNA sequencing data. In their study, only several species with multiple individuals were clustered as monophyletic and most species with multiple individuals were resolved as polyphyletic in the plastome trees. Our results referring to *Y.dezhui* and *Y.shuichengensis* were consistent with Ye et al. (2021a). In the DNA barcoding study of *Fargesia*, the nrDNA sequences showed better discriminatory power than the plastomes and some species recovered as polyphyletic in the plastome trees were resolved as monophyletic in the nrDNA phylogenies (Lv et al. 2023). The cases of *Y.dezhui* and *Y.shuichengensis* were also congruent with that of *Fargesia*. However, the specific reasons for the cytonuclear incongruence of *Y.dezhui* and *Y.shuichengensis* need to be tested within a more comprehensive sampling background in the future. On the whole, all the aforementioned studies, including our own, have demonstrated that it was better to exploit molecular markers with different inheritance patterns in discovering new species, especially those with complicated evolutionary histories.

## Supplementary Material

XML Treatment for Yushania dezhui

## ﻿Additional information

### Author ORCIDs

Yu-Xiao Zhang https://orcid.org/0000-0003-0602-3118

Chao Zhang https://orcid.org/0009-0008-7114-2459

### Data availability

All of the data that support the findings of this study are available in the main text.
